# Can the design of the instruments used for undersized osteotomies influence the initial stability of implants installed in low-density bone? An in vitro pilot study

**DOI:** 10.1371/journal.pone.0257985

**Published:** 2021-10-07

**Authors:** Márcio de Carvalho Formiga, Arthur Felipe Gehrke, João Paulo De Bortoli, Sergio Alexandre Gehrke

**Affiliations:** 1 Professor of the Implantology Program, UniSociesc, Florianópolis, Brazil; 2 Department of Mathematics, Universidade Federal de Santa Maria, Santa Maria, Brazil; 3 Professor of the Implantology Program, Associação Paulista dos Cirurgiões Dentistas—APCD, São Bernardo do Campo, Brazil; 4 Coordinator of Postgraduate Program in Implantology, Bioface Institute/UCAM/PgO, Montevideo, Uruguay; 5 Department of Biotechnology, Catholic University of Murcia, Murcia, Spain; University of Vigo, SPAIN

## Abstract

**Objectives:**

The aims of this study were to compare the initial implant stability obtained using four different osteotomy techniques in low-density synthetic bone, to evaluate the instrument design in comparison to the implant design, and to determinate a possible correlation between the insertion torque and initial stability quotient (ISQ).

**Materials and methods:**

Four groups were identified in accordance with the osteotomy technique used (n = 10 implants per group): group G1, osteotomy using the recommended drilling sequence; group G2, osteotomy using an undersized compactor drill; group G3, osteotomy using an undersized drill; and group G4, osteotomy using universal osseodensification drills. Two polyurethane blocks were used: block 1, with a medullary portion of 10 pounds per cubic foot (PCF 10) and with a 1 mm cortical portion of PCF 40, and block 2, with a medullary of PCF 15 and with a 2 mm cortical portion of PCF 40. Tapered implants of 4 mm in diameter and 11 mm in length were used. The insertion torque (IT) and ISQ were measured. The dimensions of the final instrument used in each group and the dimensions of the implant were used to calculate the total area of each part, and these data were compared.

**Results:**

Differences between the four groups were found for IT and ISQ values depending on the technique used for the osteotomy in the two synthetic bone models (p < 0.0001). All groups showed lower values of initial stability in block 1 than in block 2.

**Conclusions:**

Undersized osteotomies with instruments designed according to the implant body significantly increased the initial stability values compared to beds prepared with universal drills and using the drilling sequence standardized by the manufacturer.

## 1. Introduction

Referred to as one of the greatest advances in dentistry, dental implants have revolutionized oral rehabilitation since their inception. However, the installation of implants in areas of low bone density has been a major challenge for predictability in treatments. Thus, the search for new alternatives for these situations has been a hot topic in implant dentistry, with new micro- and macrogeometric designs of implants being presented [1–5]. In addition, changes have been proposed in the surgical technique and in the type of instrumentation used for the installation of implants; conventionally, a bone extractor (drilling) is used for creating a surgical bed with a diameter close to that of the implant that is to be installed [6–8]. Some of the main techniques proposed for the installation of implants in low-density bone are subinstrumentation, which aims to increase the bone–initial implant contact, and bone compaction through manual or rotary osteotomes, which aims to increase the bone density around the implant [9–12]. However, these techniques may sometimes fail to increase initial stability and consequently compromise secondary stability, decreasing the percentage of bone–implant contact and compromising the osseointegration process [7, 13, 14].

Initially reported by Huwais and Meyer [15], osseodensification is an osteotomy technique that preserves the bone and increases bone density by compacting the bone using instrumentation, causing the expansion of the site and increasing its density. This technique can lead to improved bone quality around implants, increasing the bone–implant contact, initial installation torque, and primary stability even in unfavorable situations. Several preclinical [15–19] and clinical [20, 21] studies have already been carried out, demonstrating the improvement of these biological factors in the peri-implant bone, which can lead to a greater probability of success in treatments. However, some implant systems use surgical instruments with format and function similar to those developed exclusively for osseodensification.

The main objective of this study was to compare the level of initial stability, measured by the insertion torque and frequency analysis by resonance (RFA), established by different instruments and/or techniques proposed to increase the initial stability for osteotomy. Implants were inserted in low-density polyurethane synthetic blocks, simulating bone with two different densities. In addition, the area of the implant body, with and without threads, in relation to the body of the last instrument used for the conformation of the osteotomy in each group, was measured and analyzed. A possible correlation between insertion torque and ISQ was analyzed.

## 2. Materials and methods

In the present study, four different groups were identified based on the received osteotomy procedures:

Group G1: the standard osteotomy sequence recommended by the implant manufacturer for the 4.0 mm conical implant—a pilot drill, followed by a 2.0 mm drill and a 3.5 mm conical drill, and ending with a 4.0 mm conical drill (Implacil, São Paulo, Brazil);
Group G2: an osteotomy sequence using a compactor instrument—a pilot drill, then a 2.0 mm drill, and then a compactor drill with counterclockwise rotation (Implacil, São Paulo, Brazil);
Group G3: an undersized osteotomy sequence—a pilot drill, then a 2.0 mm drill, and then a 3.0 mm conical drill (Implacil, São Paulo, Brazil);
Group G4: an osteotomy sequence with burs for the osseodensification of the Densah Burs system (Versah, Jackson, USA): a pilot drill, followed by tapered universal drills with incremental diameters of 2.3 mm (ref. VT1828) and 3.3 mm (ref. VT2838) with counterclockwise rotation.

**Fig 1:**
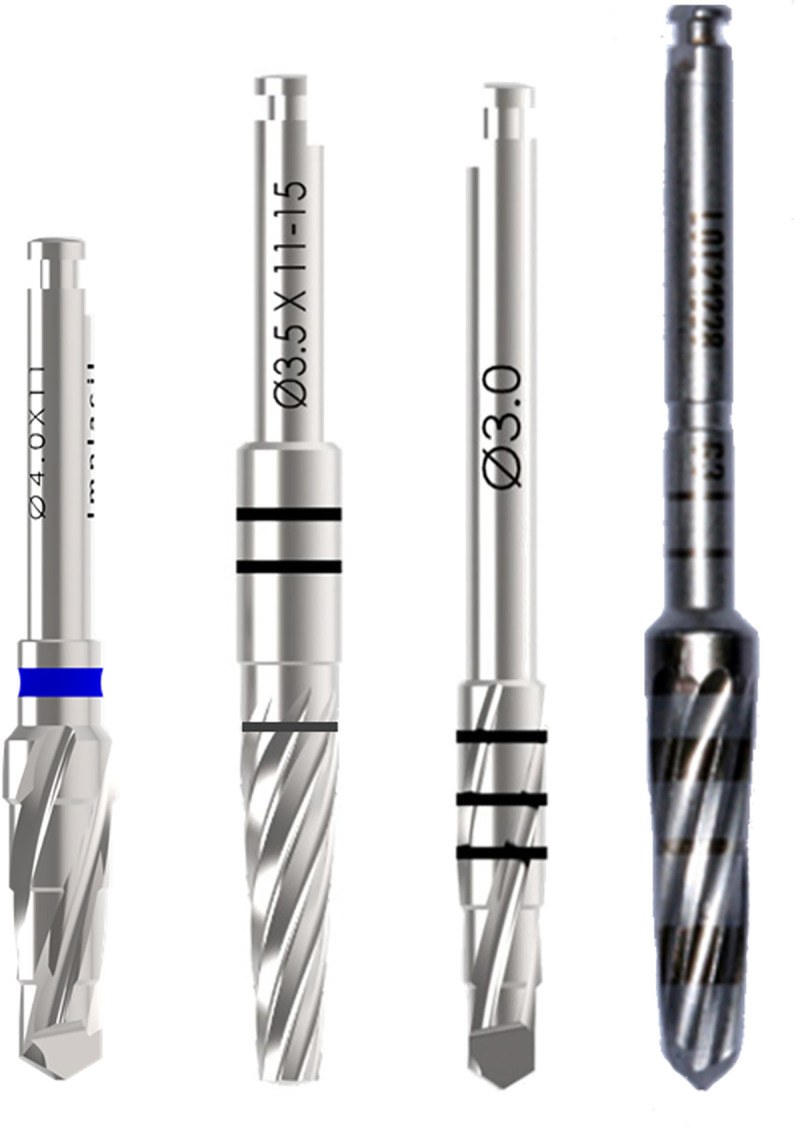
Representative image of the last instrument used in the osteotomy for each group: G1, G2, G3, and G4.

**Fig 1** shows a representative image of the last instrument used in the osteotomy for each group.

All the osteotomies were performed using an implant surgical motor (Driller BLM600, São Paulo, Brazil) with a 20:1 contra-angle at a speed of 1100 rpm.

Synthetic foam blocks of two different densities made of polyurethane foam, which is approved and recognized as a standard material for testing instruments and bone implants by the American Society for Testing and Materials [22], were used: block 1, with 10 pounds per cubic foot (PCF 10) for the medullary portion and a 1 mm cortical portion with PCF 40, and block 2, with PCF 15 for the medullary portion and a 2 mm cortical portion with PCF 40 The test blocks were purchased from the company Nacional Ossos (Jaú, Brazil). These polyurethane blocks were used to simulate low bone density (bone type 3 = block 2 and bone type 4 = block 1). The total dimensions of polyurethane blocks used were 95 mm × 45 mm × 35 mm.

**Fig 2:**
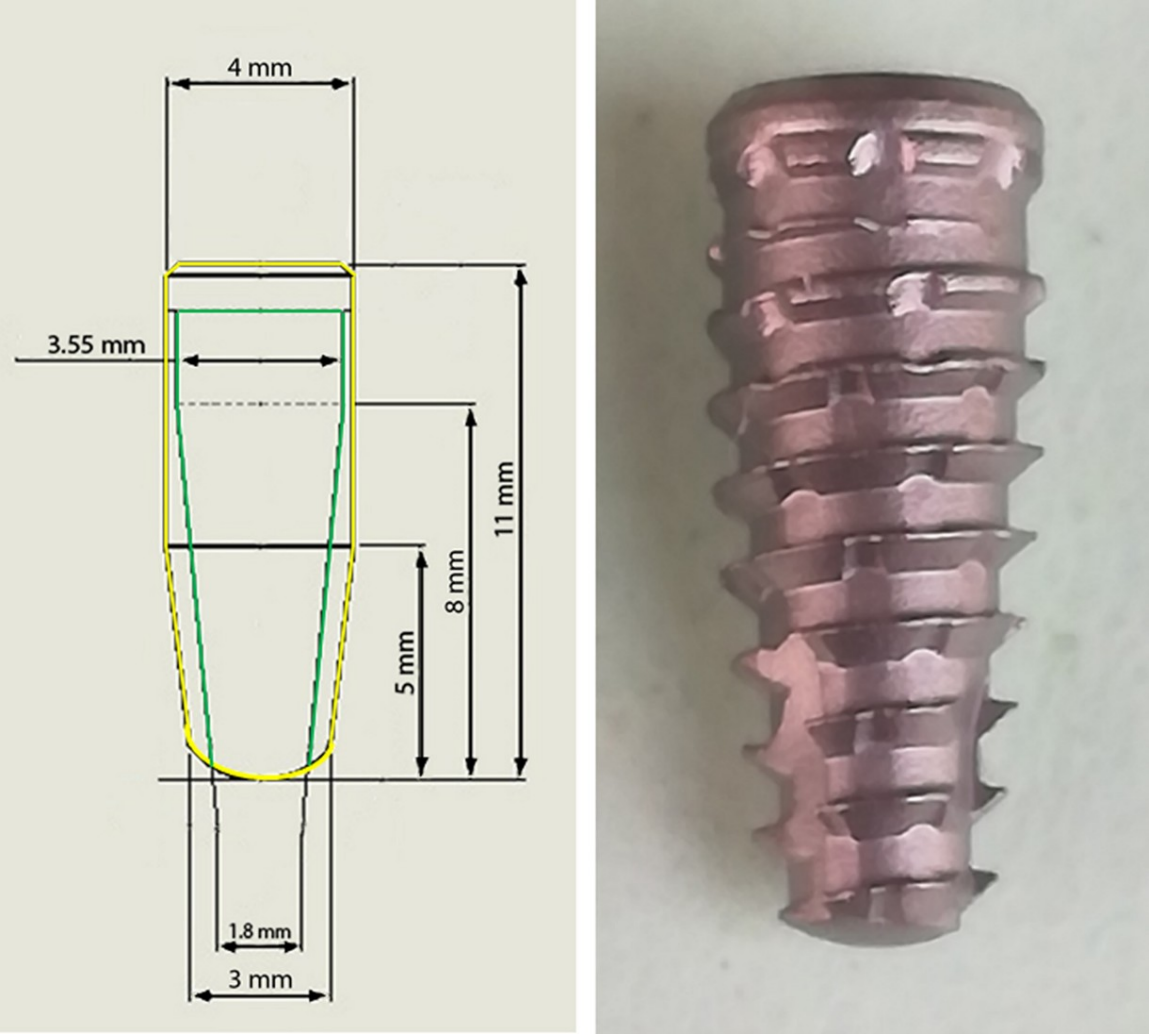
Image of the implant characteristics and design used in the present study.

After the osteotomies, 80 implants were inserted into the 2 blocks, with 20 samples per group, for a total of 10 samples from each group per block. All the implants used presented the same macrogeometric characteristics: Maestro implants (Implacil, São Paulo, Brazil) with a Morse taper connection and dimensions of 4 mm in diameter and 11 mm in length. These implants have a conical shape with trapezoidal threads and healing chambers, as shown in **Fig 2**.

The following measurements were performed: 1) the measurement of insertion torque (IT), using a digital torquemeter model TQ-680 (Instrutherm, São Paulo, Brazil), with the maximum torque measured during the insertion of the implants in the synthetic blocks until the moment when the implant platform was at the bone level; 2) measurement of the initial stability by RFA using the Osstell Mentor Device (Integration Diagnostic AB, Savadelen, Sweden), wherein immediately after the insertion of each implant, a Smart-pegs device number 16 was manually screwed to the implant. Two measurements were taken in different directions for each sample.

The total area of the implant body without the threads and the size of the total external area of the implant including the threads were calculated and compared with the size of the total area calculated for the bodies of the instruments used for the osteotomies in each group. To calculate the total area of each part, two mathematical formulas were used, with the cylindrical and conical parts of each design being considered separately. To calculate the total surface area *A* of a cylinder of diameter *d* and height *h*, the following formula was used:

$$A=\frac{1}{2}\pi dh+2\pi \frac{d^{2}}{4}.$$

For the calculation of the total surface area *A* of a conical form with diameters (*d*_*1*_, *d*_*2*_) and height *h*, the following formula was used:

$$A=\pi \frac{d_{1}^{2}+d_{2}^{2}}{4}+\pi \frac{d_{1}+d_{2}}{2}\sqrt{h^{2}+{\left(\frac{d_{1}-d_{2}}{2}\right)}^{2}}.$$

**Fig 3:**
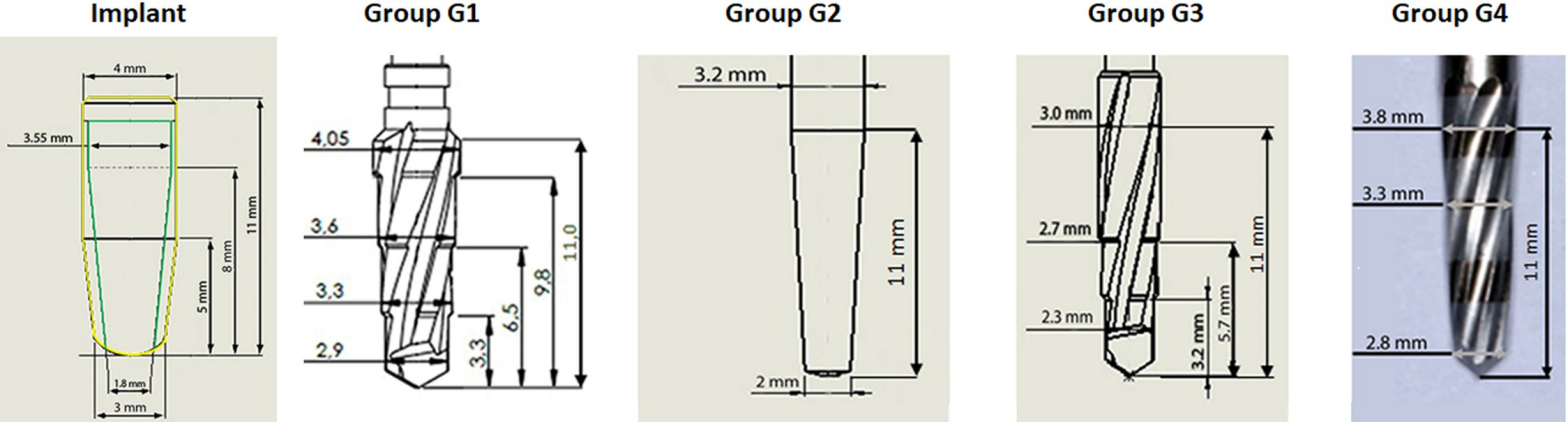
Image of the dimensions of each part used in the study. The green line corresponds to the implant body without the threads, and the yellow line corresponds to the external limits of the implant with the threads.

Both formulas were used according to Spivak [23]. **Fig 3** shows the values considered for the calculation according to the geometry of each part.

The D’Agostino and Pearson omnibus normality test was applied to verify the normality of the data. One-way ANOVA was applied to detect statistical differences in the data for different groups in the same bone block. Then, Bonferroni’s multiple comparison test was used to statistically compare the data between the groups in the same bone block model. Pearson’s correlation test was used to evaluate the correlation between insertion torque and initial stability quotient in all proposed groups. The GraphPad Prism program version 5.01 for Windows (GraphPad Software, San Diego, California, USA) was used to perform all statistical analyses. Results were considered statistically significant when the p-value <0.05.

## 3. Results

In both blocks, normality was detected in the distribution of data measured for the insertion torque and initial stability quotient (ISQ).

**Fig 4:**
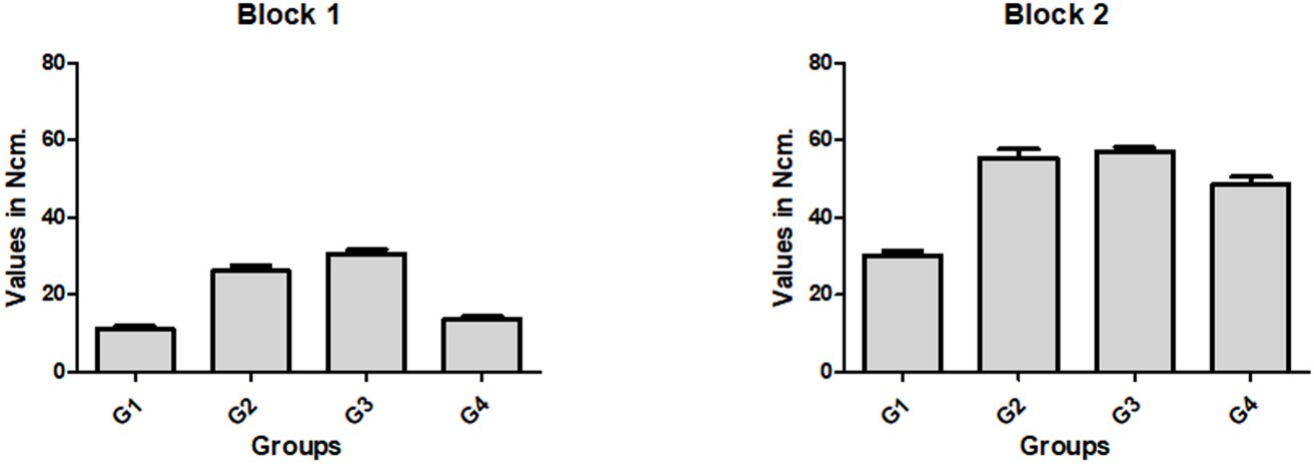
Bar graphs of the mean and standard deviation of insertion torque values measured for each group in both synthetic bone blocks used (blocks 1 and 2).

For all four groups within the same synthetic bone model, block 2 presented a 134.3% higher average insertion torque and a 39.2% higher average ISQ compared with block 1. Furthermore, in both blocks, the four groups presented statistically significant (p < 0.0001) differences in insertion torque. The bar graph in **Fig 4** shows the data values (mean and standard deviation) for each group in the two blocks.

**Table 1** shows a comparative statistical analysis of the insertion torque between the four groups in both block models tested.

**Table 1:** Bonferroni’s multiple comparison test to compare insertion torque values between the groups in the two synthetic bone blocks.

		Block 1			Block 2	
Group Comparison	Mean of Diff.	*p-*value	95% CI	Mean of Diff.	*p-*value	95% CI
**G1 vs. G2**	-14.90	0.0001*	-19.16 to -10.64	-25.40	0.0001*	-32.28 to -18.52
**G1 vs. G3**	-19.40	0.0001*	-23.66 to -15.14	-26.90	0.0001*	-33.78 to -20.02
**G1 vs. G4**	-2.500	0.0241*	-6.760 to 1.760	-18.40	0.0001*	-25.28 to -11.52
**G2 vs. G3**	-4.500	0.0532	-8.76 to -0.2403	-1.500	0.8642	-8.376 to 5.376
**G2 vs. G4**	12.40	0.0002*	8.140 to 16.66	7.000	0.0498*	0.1235 to 13.88
**G3 vs. G4**	16.90	0.0002*	12.64 to 21.16	8.500	0.0084*	1.624 to 15.38

Diff. = Differences

* with difference statistical (*p* < 0.05); CI = Confidence Interval.

**Fig 5:**
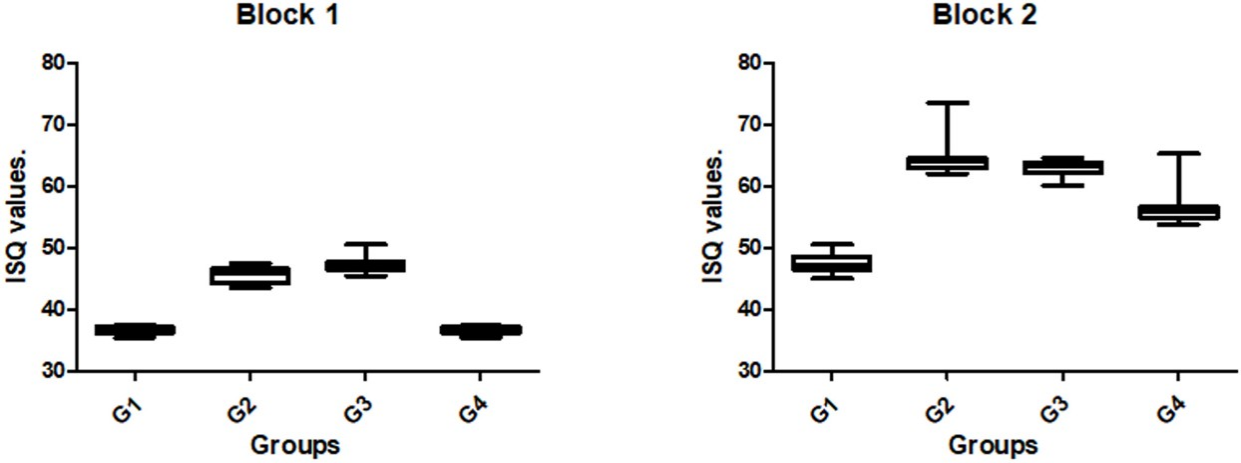
Box plot graphs of ISQ data for each group in both synthetic bone blocks used (blocks 1 and 2).

The ISQ values measured of the implants presented different values for the proposed groups in both blocks, with statistically significant differences (p < 0.0001). The bar graph in **Fig 5** shows the data values (mean and standard deviation) for each group in the two blocks.

**Table 2** shows a comparative statistical analysis of the ISQ values between the four groups in both block models tested.

**Table 2:** Bonferroni’s multiple comparison test to compare Implant Stability Quotients (ISQs) between the groups in the two synthetic bone blocks.

		Block 1			Block 2	
Group Comparison	Mean of Diff.	*p-*value	95% CI	Mean of Diff.	*p-*value	95% CI
**G1 vs. G2**	-9.100	0.0002*	-10.44 to -7.762	-17.00	0.0002*	-20.13 to -13.87
**G1 vs. G3**	-10.70	0.0002*	-12.04 to -9.362	-15.34	0.0002*	-18.47 to -12.21
**G1 vs. G4**	0.05000	0.8414	-1.288 to 1.388	-8.960	0.0002*	-12.09 to -5.835
**G2 vs. G3**	-1.600	0.0218*	-2.938 to -0.2622	1.660	0.2189	-1.465 to 4.785
**G2 vs. G4**	9.150	0.0002*	7.812 to 10.49	8.040	0.0021*	4.915 to 11.17
**G3 vs. G4**	10.75	0.0002*	9.412 to 12.09	6.380	0.0028*	3.255 to 9.505

Diff. = differences

* = statistically significant difference (*p* < 0.05); CI = confidence interval.

No correlation was detected between the insertion torque and ISQ values in the proposed groups. **Table 3** shows the analysis results for each group in both synthetic bone blocks.

**Table 3:** Pearson correlation analysis with Pearson-*r*, *p*-value, and *r*-square between insertion torque and initial stability quotient of all groups in both synthetic bone blocks.

Group	Block 1	Block 2
**G1**	r = 0.4697/p = 0.1708/r^2^ = 0.2206	r = -0.1211/p = 0.7389/r^2^ = 0.01467
**G2**	r = 0.1454/p = 0.6886/r^2^ = 0.2113	r = 0.02120/p = 0.9537/r^2^ = 0.00045
**G3**	r = 0.2342/p = 0.5149/r^2^ = 0.05485	r = -0.2672/p = 0.4555/r^2^ = 0.07137
**G4**	r = 0.4236/p = 0.2225/r^2^ = 0.1795	r = 0.6188/p = 0.0565/r^2^ = 0.3829

r = correlation coefficient; *p* = *p*-value.

The area of the implant body (without the threads) was calculated as 159.9 mm^2^, and the total area calculated with the threads was 175 mm^2^. The calculations of the areas of the osteotomy instruments corresponding to each group and the difference between the two area values calculated for the implant are presented in **Table 4**.

**Table 4:** Calculated area of the last instrument used in each group and the difference in relation of the implant body area (without threads) and the external area of the implant with the threads.

Group	Total area	Difference 1	Difference 2
**G1**	168.5 mm^2^	8.6 mm^2^	-6.5 mm^2^
**G2**	101.2 mm^2^	-58.7 mm^2^	-73.8 mm^2^
**G3**	128.2 mm^2^	-31.7 mm^2^	-46.8 mm^2^
**G4**	131.7 mm^2^	-28.2 mm^2^	-43.3 mm^2^

Difference 1 = total area of the instrument–total area of the implant body; difference 2 = total area of the instrument–total area of the implant with the threads.

## 4. Discussion

In this pilot in vitro study, we compared four osteotomy techniques (one using the recommended drill sequence for the implant design and three undersized sequences) used for implant bed preparation in low-density synthetic polyurethane bone blocks. Moreover, the areas of the implant body, the implant body plus the external threads, and the relevant instruments were calculated to evaluate the relation of the area to the initial implant stability (IT and ISQ values). The results showed that in block 1 (1 mm of cortical PCF 40 and medullary portion of PCF 10), which simulated type 4 bone, even when using undersized osteotomy, the initial implant stability (IT and ISQ values) was quite low for all groups in comparison with that in block 2 (2 mm of cortical PCF 40 and medullary portion of PCF 15), which simulated type 3 bone. Additionally, by analyzing the calculated areas of each part used (implant and instruments) in relation to the initial stability data obtained, we verified that the design relationship between the parts had a significant influence on the results.

The installation of implants in low-bone-density areas can cause problems for good initial stability, which is considered an important condition to obtain adequate osseointegration [24–29]. Initial stability is evaluated by the presence of micromovements of implants immediately after their insertion into the bone tissue [30, 31]. According to the majority of studies related to the initial stability of implants, the most frequently used methods for measuring initial stability are the measurement of insertion torque and analysis by resonance frequency [32–34]; we selected these methods for our study. The substrate used for the tests, synthetic polyurethane blocks, was previously used in our group of studies [35] and also by other researchers [31, 36–38], who considered it valid for assessing the initial stability and insertion torque of implants because of its similarity with human bone tissue.

The insertion torque is the first clinical information obtained during implant installation in relation to initial stability. In bone types 1 and 2, which present high density, an adequate initial implant stability with a high insertion torque is easily obtained by using the drilling sequence recommended for osteotomy for the implant design used [39]. However, bone types 3 and 4, which have a low density; high values for insertion torque; and, consequently, adequate initial stability, were shown to be more difficult to obtain using the recommended drilling sequence proposed by the manufacturer [40], corroborating our results in the present study. Thus, changes in the bed preparation technique used for implant placement were tested, obviously all with underdimensioning in relation to the diameter of the implant to be installed. This type of modification in the technique (undersized beds) has been extensively tested and described in the literature [41–43], giving rise to systems considered universal for this purpose. However, our results for the group for which the bed was prepared using a universal system for osteotomy (group G4) in low-density bone (block 1) showed values much lower than those shown for groups G2 and G3 (127.2% higher on average), where undersized instruments from the same manufacturer for the implant were used. These results corroborate the findings recently published by Delgado-Ruiz and collaborators [44].

Comparing the results obtained in each block used, block 2 showed greater values of initial stability compared to those found for block 1. The insertion torque was 134.3% greater, and ISQ was 39.2% greater. These results are in agreement with other studies that showed that the bone density [40, 45], and especially the thickness of the cortical portion [46, 47], directly influence the initial implant stability, regardless of the shape of the implant or the technique used to prepare the bed. However, the macrogeometry of the implant can also significantly influence the initial stability [35, 47, 48]; therefore, in our study, the same implant model was used for all proposed groups.

In terms of the initial stability measured using the RFA method (i.e., ISQ), the results showed different values depending on the density of the blocks, which corroborated the results of other similar studies [42–51]. In block 1 (simulating bone type 4), all groups presented mean values for ISQ below 50, which is considered low for initial implant stability [52] and can increase the risk of failure (poor osseointegration) [43]. For this density, the highest ISQ values were obtained with the undersized osteotomies used for groups G2 and G3; these values were an average of 27% higher in comparison to those used for groups G1 and G4, corroborating the results obtained for insertion torque. In block 2 (simulating bone type 3), the ISQ values were an average of 22.5% higher in groups G2 and G3 than those obtained in groups G1 and G4. However, it is important to understand that RFA analysis indicates the absence of mobility in the installed implant and not the bone quantity at the implant–bone interface [53].

Lages and collaborators reported that IT and RFA were independent and incomparable methods for measuring primary stability and alerted clinicians to the importance of defining only one method for assessing implant stability [32]. As reported in other studies, no correlation was found between IT and RFA values [9]. These results show that analyses of the frequency of resonance in dental implants should be carefully evaluated, as several factors can influence the measured values.

Our results showed that the implant design compared to the instrument design used for the osteotomy is an important factor to consider. In both synthetic bone densities tested, the best results for initial stability were obtained in groups G2 and G3, in which the instrument design considered the design of the implant body. Although the relation of the calculated area of the instrument within the bone bed and the implant body with and without the threads presented similar values for groups G3 and G4, the IT and RFA results were much higher for group G3, which led us to conclude that the difference lay in the design of the dimensions of each part used.

As a limitation of the present study, we can cite the homogeneity of the blocks used, which rarely occurs under clinical conditions. The used blocks were selected with two thicknesses of cortical lamina, which is variable in clinical situations. Still, all maneuvers were performed without clinical conditions. The lack of an organic portion, present in bone but not in the synthetic polyurethane blocks used here, is another important factor that must be considered in regard to the obtained results. Future studies with other implant models, especially those with designs similar to the instrument used in the G4 group, would help to corroborate our results.

## 5. Conclusions

From the results obtained, and within the limitations of the present in vitro study, we observed that to increase the initial stability values of the implants, undersized osteotomy should be performed with an instrument designed according to the dimensions of the implant body. The preparation of the implant bed with universal osseodensification instruments led to a low initial stability of the implants, especially in lower-density bone (PCF 10), in comparison to the stability achieved through preparation with undersized instruments that were designed for the implant model used.
